# 2,4-Di­chloro-6-{*N*-[2-(tri­fluoro­meth­yl)phen­yl]carboximido­yl}phenol

**DOI:** 10.1107/S2414314624010745

**Published:** 2024-11-14

**Authors:** Zizipho Xantini, Alfred Muller, Koop Lammertsma

**Affiliations:** a Department of Chemical Sciences, University of Johannesburg, 2006, South Africa; University of Kentucky, USA

**Keywords:** crystal structure, Schiff base, tri­fluoro­methyl­aniline, di­chloro­salicyl­aldehyde, ortho­rhom­bic

## Abstract

The title compound, C_14_H_8_Cl_2_F_3_NO, was synthesized by the condensation between tri­fluoro­methyl­aniline and di­chloro­salicyl­aldehyde by nucleophilic addition, forming a hemiaminal, followed by a dehydration to generate an imine. The compound crystallizes in an ortho­rhom­bic *Pbca* (*Z* = 8) space group with a dihedral angle of 44.70 (5)° between the two aromatic rings. In the crystal, the mol­ecules pack together to form a zigzag pattern along the *c* axis.

## Abstract 2

The title compound was synthesized by the condensation between tri­fluoro­methyl­aniline and di­chloro­salicyl­aldehyde by nucleophilic addition, forming a hemiaminal, followed by a dehydration to generate an imine. The compound crystallizes in an ortho­rhom­bic *Pbca* (*Z* = 8) space group with a dihedral angle of 44.70 (5)° between the two aromatic rings. In the crystal, the mol­ecules pack together to form a zigzag pattern along the *c* axis.

## Graphical abstract

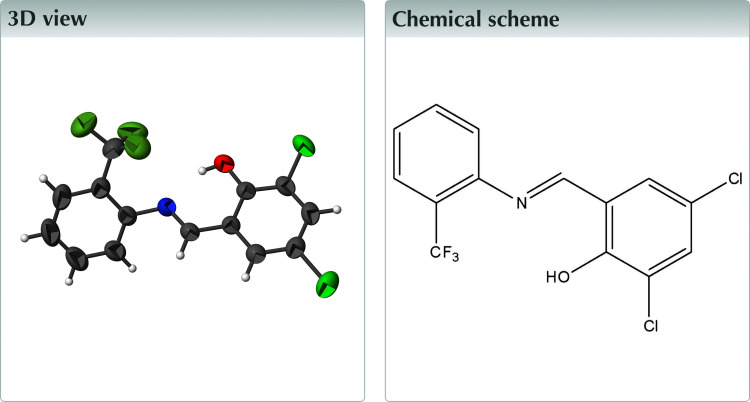

## Structure description

Schiff base compounds have been synthesized since their discovery in 1864 (Tidwell, 2008 ▸), and have shown good viability as ligands in Schiff base–metal complexes (Ngan *et al.*, 2011 ▸). Their versatility is attributed to the extensive range of potential ligand structures, which are dependent on the selection of aldehydes and amines (More *et al.*, 2019 ▸) as starting reagents, with possibilities including bidentate, tridentate and tetra­dentate ligands. The synthesis typically involves condensation of primary amines and carbonyl compounds by nucleophilic addition, forming a hemiaminal, followed by a dehydration to generate an imine (Tovrog *et al.*, 1976 ▸; Maihub *et al.*, 2013 ▸; da Silva *et al.*, 2011 ▸). The generated azomethine (C=N) dominates the properties of Schiff bases as the most important part in the Schiff base body (Maihub *et al.*, 2013 ▸), and these have shown to be important for a wide range of applications in biological applications such as anti- bacterial and anti-fungal activities (Tovrog *et al.*, 1976 ▸; Dhar & Taploo, 1982 ▸). As part of ongoing research in our group, we report here the synthesis and a crystal structure of a Schiff base ligand derived from di­chloro­salicyl­aldehyde and 2-tri­fluoro­methyl­aniline.

**Figure 1:**
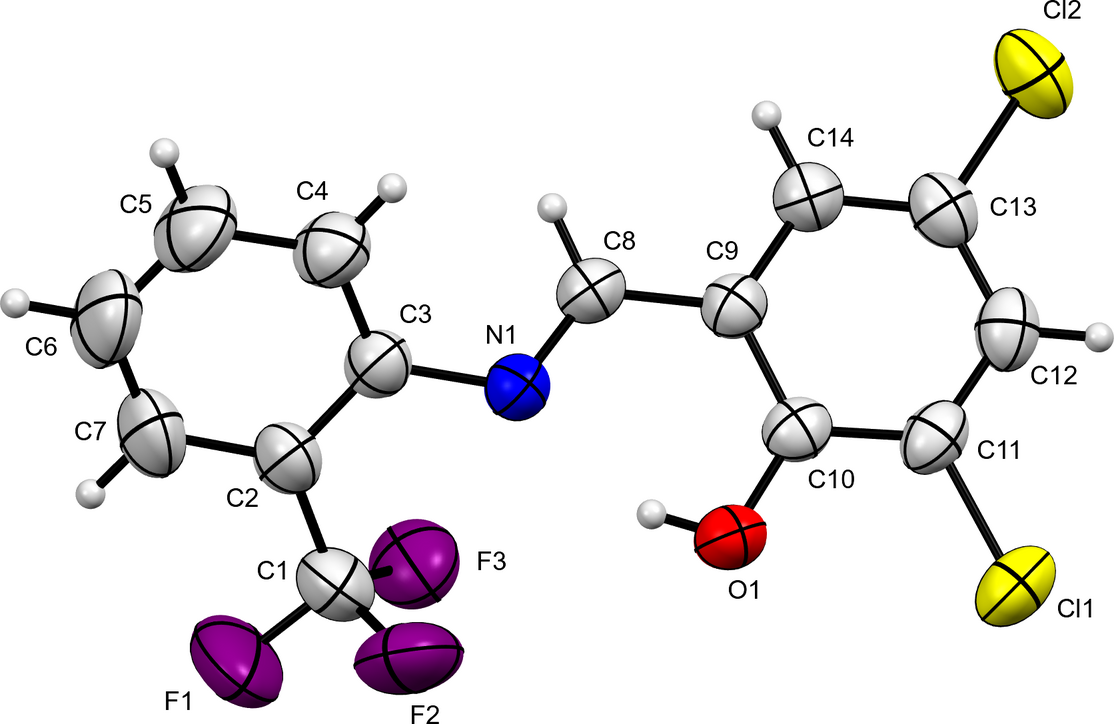
An ellipsoid plot of the title compound with the atom-labelling scheme and displacement ellipsoids drawn at 50% probability level. H atoms are shown as small circles of arbitrary radius.

**Figure 2:**
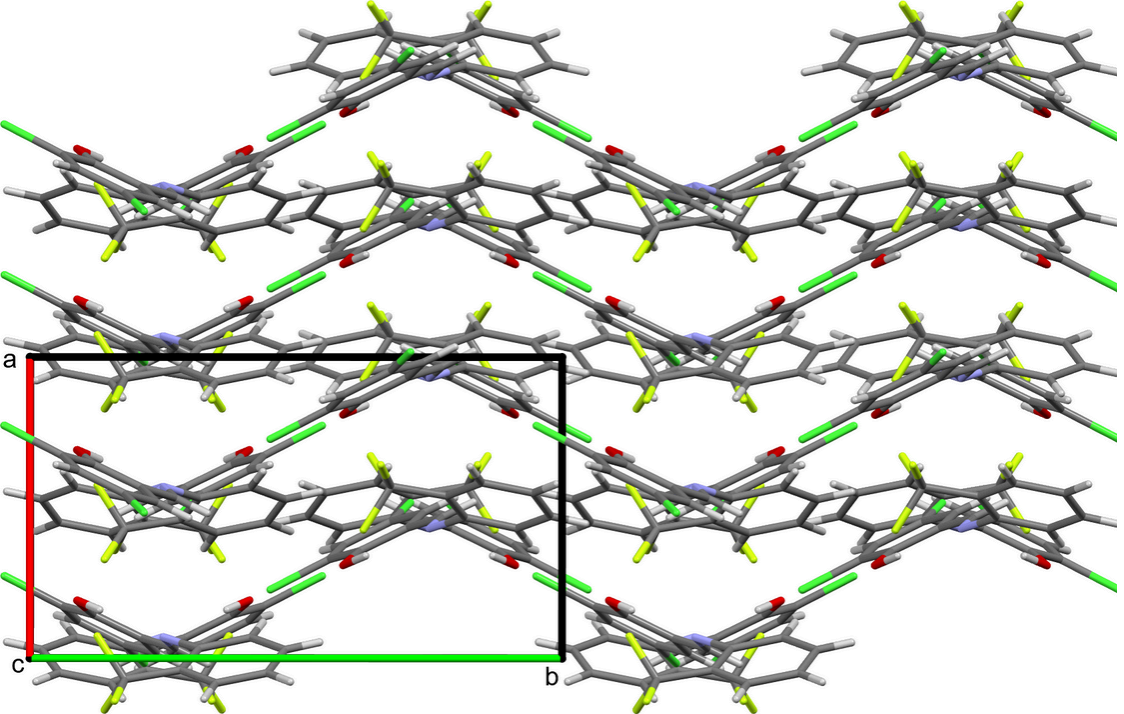
The crystal packing of the title compound, showing the layered packing arrangement, viewed along the *c* axis.

The title compound (Fig. 1 ▸) crystallizes in the *Pbca* (*Z* = 8) space group with mol­ecules on general positions. The bond length for the azomethine group [N1—C8 = 1.274 (2) Å] suggest the presence of double-bond character as anti­cipated. The dihedral angle of 44.70 (5)° between the aromatic rings is predominantly attributed to the intra­molecular O—H⋯N and inter­molecular C—H⋯O hydrogen bonding (see Table 1 ▸). The mol­ecules pack together to form a zigzag pattern along the *c* axis (see Fig. 2 ▸).

## Synthesis and crystallization

2-Tri­fluoro­methyl­aniline (1.0740 g, 20 mmol) and di­chloro­salicyl­aldehyde (1.2662 g, 20 mmol) were refluxed in 10 ml of methanol overnight to give an orange solution. The volume of methanol was reduced, which resulted in an orange precipitate. Orange crystals were obtained by slow evaporation of a methanol solution. Yield 70.16%, 1.5578 g, 4.679 mmol; m.p. 140–143°C; IR (KBr, cm^−1^): ν (Ar—OH) 3086; ν (C=N) 1612; ν (C—O) 1273. ^1^H NMR (DMSO-*d*_6_ in p.p.m, *J* in Hz): δ = 13.49 [1H (*s*), OH], 9.05 [1H (*s*), H5], 7.85 [2H (*d*, *J* = 10), H1, H2,], 7.81 [2H (*s*), H1,H8], 7.67 [1H, (*d*, *J* = 10), H7], 7.57 [1H, (*t*, *J* = 5)].

## Refinement

Crystal data, data collection and structure refinement details are summarized in Table 2 ▸.

## Supplementary Material

Crystal structure: contains datablock(s) I. DOI: 10.1107/S2414314624010745/pk4045sup1.cif

Structure factors: contains datablock(s) I. DOI: 10.1107/S2414314624010745/pk4045Isup2.hkl

Supporting information file. DOI: 10.1107/S2414314624010745/pk4045Isup3.cml

CCDC reference: 2400964

Additional supporting information:  crystallographic information; 3D view; checkCIF report

## Figures and Tables

**Table 1:** Hydrogen-bond geometry (Å, °)

*D*—H⋯*A*	*D*—H	H⋯*A*	*D*⋯*A*	*D*—H⋯*A*
O1—H1⋯N1	0.82 (2)	1.86 (2)	2.6111 (18)	152 (2)
C14—H14⋯O1^i^	0.93	2.67	3.515 (2)	151

Symmetry code: (i) 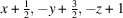.

**Table 2:** Experimental details

Crystal data	
Chemical formula	C_14_H_8_Cl_2_F_3_NO
*M*_r_	334.11
Crystal system, space group	Orthorhombic, *Pbca*
Temperature (K)	293
*a*, *b*, *c* (Å)	7.8436 (3), 13.8612 (4), 25.8433 (7)
*V* (Å^3^)	2809.73 (15)
*Z*	8
Radiation type	Mo *K*α
μ (mm^−1^)	0.49
Crystal size (mm)	0.38 × 0.22 × 0.14

Data collection	
Diffractometer	Bruker APEXII CCD
Absorption correction	Multi-scan (*SADABS*; Krause *et al.*, 2015 ▸)
*T*_min_, *T*_max_	0.680, 0.746
No. of measured, independent and observed [*I* > 2σ(*I*)] reflections	27956, 3353, 2679
*R*_int_	0.040
(sin θ/λ)_max_ (Å^−1^)	0.658

Refinement	
*R*[*F*^2^ > 2σ(*F*^2^)], *wR*(*F*^2^), *S*	0.036, 0.111, 1.06
No. of reflections	3353
No. of parameters	194
H-atom treatment	H atoms treated by a mixture of independent and constrained refinement
Δρ_max_, Δρ_min_ (e Å^−3^)	0.20, −0.36

Computer programs: *APEX2* and *SAINT* (Bruker, 2010 ▸), *SHELXT* (Sheldrick, 2015*a* ▸), *Mercury* (Macrae *et al.*, 2020 ▸), *SHELXL* (Sheldrick, 2015*b* ▸) and *publCIF* (Westrip, 2010 ▸).

## full crystallographic data

### 2,4-Dichloro-6-{N-[2-(trifluoromethyl)phenyl]carboximidoyl}phenol Crystal data

**Table:**

C_14_H_8_Cl_2_F_3_NO	*D*_x_ = 1.580 Mg m^−^^3^
*M**_r_* = 334.11	Mo *K*α radiation, λ = 0.71073 Å
Orthorhombic, *P**b**c**a*	Cell parameters from 9920 reflections
*a* = 7.8436 (3) Å	θ = 3.0–27.7°
*b* = 13.8612 (4) Å	µ = 0.49 mm^−^^1^
*c* = 25.8433 (7) Å	*T* = 293 K
*V* = 2809.73 (15) Å^3^	Block, yellow
*Z* = 8	0.38 × 0.22 × 0.14 mm
*F*(000) = 1344	

### 2,4-Dichloro-6-{N-[2-(trifluoromethyl)phenyl]carboximidoyl}phenol Data collection

**Table:**

Bruker APEXII CCD diffractometer	2679 reflections with *I* > 2σ(*I*)
φ and ω scans	*R*_int_ = 0.040
Absorption correction: multi-scan (*SADABS*; Krause *et al*., 2015)	θ_max_ = 27.9°, θ_min_ = 1.6°
*T*_min_ = 0.680, *T*_max_ = 0.746	*h* = −9→10
27956 measured reflections	*k* = −18→14
3353 independent reflections	*l* = −33→33

### 2,4-Dichloro-6-{N-[2-(trifluoromethyl)phenyl]carboximidoyl}phenol Refinement

**Table:**

Refinement on *F*^2^	0 restraints
Least-squares matrix: full	Hydrogen site location: mixed
*R*[*F*^2^ > 2σ(*F*^2^)] = 0.036	H atoms treated by a mixture of independent and constrained refinement
*wR*(*F*^2^) = 0.111	*w* = 1/[σ^2^(*F*_o_^2^) + (0.0542*P*)^2^ + 0.6574*P*] where *P* = (*F*_o_^2^ + 2*F*_c_^2^)/3
*S* = 1.06	(Δ/σ)_max_ = 0.001
3353 reflections	Δρ_max_ = 0.20 e Å^−^^3^
194 parameters	Δρ_min_ = −0.36 e Å^−^^3^

### 2,4-Dichloro-6-{N-[2-(trifluoromethyl)phenyl]carboximidoyl}phenol Special details

**Table:**

Experimental. Crystallographic data was collected on a Bruker DUO *APEX* II diffractometer (Bruker, 2010). The intensity data were obtained and adjusted using the *SAINT* software (Bruker, 2010). The correction of the collected intensities for absorption was done using *SADABS* (Krause *et al.*, 2015). The structure was solved using *SHELXT* (Sheldrick, 2015*a*). The refinement of the structure was done using *SHELXL* (Sheldrick, 2015*b*) with the *X-SEED* (Barbour, 2020) software interface using anisotropic models for non-hydrogen atoms. *Mercury* (Macrae *et al.*, 2020) was used to prepare the figures for publication.
Geometry. All esds (except the esd in the dihedral angle between two l.s. planes) are estimated using the full covariance matrix. The cell esds are taken into account individually in the estimation of esds in distances, angles and torsion angles; correlations between esds in cell parameters are only used when they are defined by crystal symmetry. An approximate (isotropic) treatment of cell esds is used for estimating esds involving l.s. planes.

### 2,4-Dichloro-6-{N-[2-(trifluoromethyl)phenyl]carboximidoyl}phenol Fractional atomic coordinates and isotropic or equivalent isotropic displacement parameters (Å^2^)

**Table:**

	*x*	*y*	*z*	*U*_iso_*/*U*_eq_	
Cl1	0.22744 (7)	0.45151 (3)	0.52669 (2)	0.07023 (17)	
Cl2	0.51694 (7)	0.71474 (4)	0.65169 (2)	0.07656 (18)	
O1	0.30841 (17)	0.58814 (9)	0.44463 (5)	0.0565 (3)	
N1	0.43077 (18)	0.75064 (9)	0.40953 (5)	0.0490 (3)	
F1	0.6420 (3)	0.65886 (12)	0.27056 (5)	0.1294 (7)	
C1	0.5703 (3)	0.67847 (16)	0.31575 (7)	0.0737 (6)	
C2	0.5393 (3)	0.78362 (13)	0.32338 (7)	0.0587 (4)	
C3	0.4678 (2)	0.81737 (11)	0.36918 (6)	0.0508 (4)	
C4	0.4317 (3)	0.91504 (13)	0.37471 (8)	0.0655 (5)	
H4	0.380492	0.937641	0.404823	0.079*	
C5	0.4722 (3)	0.97866 (16)	0.33510 (10)	0.0846 (7)	
H5	0.447098	1.043908	0.338600	0.101*	
C6	0.5491 (4)	0.94580 (17)	0.29087 (9)	0.0917 (8)	
H6	0.578551	0.989010	0.264838	0.110*	
C7	0.5829 (3)	0.84888 (17)	0.28488 (8)	0.0796 (6)	
H7	0.635311	0.827058	0.254796	0.096*	
C8	0.4679 (2)	0.77357 (11)	0.45590 (6)	0.0476 (3)	
H8	0.518662	0.833044	0.462209	0.057*	
C9	0.43343 (19)	0.70977 (10)	0.49926 (6)	0.0448 (3)	
C10	0.35574 (19)	0.61936 (10)	0.49151 (6)	0.0459 (3)	
C11	0.3277 (2)	0.56153 (11)	0.53473 (7)	0.0509 (4)	
C12	0.3767 (2)	0.58980 (12)	0.58379 (7)	0.0556 (4)	
H12	0.357636	0.549712	0.612062	0.067*	
F2	0.4265 (2)	0.62654 (10)	0.31795 (6)	0.1064 (5)	
F3	0.6717 (2)	0.63993 (9)	0.35177 (5)	0.0902 (4)	
C14	0.4823 (2)	0.73832 (12)	0.54898 (6)	0.0494 (4)	
H14	0.533728	0.798015	0.554050	0.059*	
C13	0.4542 (2)	0.67828 (13)	0.59027 (6)	0.0535 (4)	
H1	0.340 (3)	0.6303 (17)	0.4244 (9)	0.076 (7)*	

### 2,4-Dichloro-6-{N-[2-(trifluoromethyl)phenyl]carboximidoyl}phenol Atomic displacement parameters (Å^2^)

**Table:**

	*U*^11^	*U*^22^	*U*^33^	*U*^12^	*U*^13^	*U*^23^
Cl1	0.0718 (3)	0.0469 (2)	0.0920 (4)	−0.00955 (19)	0.0038 (2)	0.0095 (2)
Cl2	0.0816 (4)	0.1008 (4)	0.0473 (3)	0.0027 (3)	−0.0062 (2)	−0.0007 (2)
O1	0.0660 (7)	0.0459 (6)	0.0575 (7)	−0.0080 (6)	−0.0031 (6)	−0.0028 (5)
N1	0.0545 (8)	0.0446 (6)	0.0480 (7)	−0.0019 (6)	−0.0011 (6)	0.0014 (5)
F1	0.211 (2)	0.1037 (11)	0.0738 (9)	0.0138 (13)	0.0464 (11)	−0.0189 (8)
C1	0.0995 (16)	0.0697 (12)	0.0520 (10)	−0.0034 (12)	0.0069 (10)	−0.0094 (9)
C2	0.0709 (11)	0.0610 (10)	0.0441 (9)	−0.0053 (9)	−0.0093 (8)	0.0030 (7)
C3	0.0542 (9)	0.0494 (8)	0.0488 (9)	−0.0043 (7)	−0.0089 (7)	0.0042 (7)
C4	0.0782 (12)	0.0506 (9)	0.0677 (11)	0.0024 (9)	−0.0076 (9)	0.0051 (8)
C5	0.1106 (18)	0.0551 (10)	0.0880 (16)	0.0004 (12)	−0.0164 (14)	0.0210 (10)
C6	0.125 (2)	0.0807 (15)	0.0689 (13)	−0.0138 (14)	−0.0142 (14)	0.0330 (12)
C7	0.1047 (17)	0.0875 (15)	0.0466 (10)	−0.0091 (13)	−0.0052 (10)	0.0140 (9)
C8	0.0481 (8)	0.0420 (7)	0.0527 (9)	−0.0033 (6)	−0.0003 (7)	−0.0001 (6)
C9	0.0433 (7)	0.0433 (7)	0.0479 (8)	0.0015 (6)	0.0012 (6)	0.0009 (6)
C10	0.0429 (8)	0.0419 (7)	0.0529 (8)	0.0041 (6)	0.0009 (6)	−0.0009 (6)
C11	0.0454 (8)	0.0411 (7)	0.0661 (10)	0.0025 (6)	0.0051 (7)	0.0070 (7)
C12	0.0518 (9)	0.0575 (9)	0.0574 (9)	0.0073 (7)	0.0062 (7)	0.0145 (7)
F2	0.1260 (13)	0.0786 (8)	0.1145 (12)	−0.0282 (8)	−0.0055 (9)	−0.0313 (8)
F3	0.1110 (11)	0.0675 (7)	0.0922 (9)	0.0197 (7)	0.0019 (8)	0.0058 (6)
C14	0.0476 (8)	0.0490 (8)	0.0514 (9)	0.0005 (7)	−0.0007 (7)	−0.0004 (7)
C13	0.0509 (9)	0.0639 (10)	0.0457 (8)	0.0070 (7)	−0.0002 (7)	0.0011 (7)

### 2,4-Dichloro-6-{N-[2-(trifluoromethyl)phenyl]carboximidoyl}phenol Geometric parameters (Å, º)

**Table:**

Cl1—C11	1.7286 (16)	C5—C6	1.370 (4)
Cl2—C13	1.7369 (18)	C5—H5	0.9300
O1—C10	1.3389 (19)	C6—C7	1.378 (3)
O1—H1	0.82 (2)	C6—H6	0.9300
N1—C8	1.274 (2)	C7—H7	0.9300
N1—C3	1.424 (2)	C8—C9	1.453 (2)
F1—C1	1.324 (2)	C8—H8	0.9300
C1—F3	1.336 (3)	C9—C14	1.398 (2)
C1—F2	1.339 (3)	C9—C10	1.408 (2)
C1—C2	1.491 (3)	C10—C11	1.392 (2)
C2—C7	1.388 (3)	C11—C12	1.382 (3)
C2—C3	1.391 (3)	C12—C13	1.379 (3)
C3—C4	1.391 (2)	C12—H12	0.9300
C4—C5	1.388 (3)	C14—C13	1.371 (2)
C4—H4	0.9300	C14—H14	0.9300
			
C10—O1—H1	105.2 (16)	C6—C7—H7	119.7
C8—N1—C3	118.70 (14)	C2—C7—H7	119.7
F1—C1—F3	106.3 (2)	N1—C8—C9	122.10 (14)
F1—C1—F2	106.53 (19)	N1—C8—H8	119.0
F3—C1—F2	104.88 (18)	C9—C8—H8	119.0
F1—C1—C2	112.73 (18)	C14—C9—C10	120.08 (14)
F3—C1—C2	113.33 (17)	C14—C9—C8	119.06 (14)
F2—C1—C2	112.5 (2)	C10—C9—C8	120.84 (14)
C7—C2—C3	119.36 (18)	O1—C10—C11	119.74 (14)
C7—C2—C1	120.17 (18)	O1—C10—C9	122.44 (14)
C3—C2—C1	120.47 (16)	C11—C10—C9	117.82 (14)
C4—C3—C2	119.81 (16)	C12—C11—C10	121.95 (15)
C4—C3—N1	121.04 (16)	C12—C11—Cl1	119.14 (13)
C2—C3—N1	119.14 (15)	C10—C11—Cl1	118.90 (13)
C5—C4—C3	119.7 (2)	C13—C12—C11	119.09 (15)
C5—C4—H4	120.1	C13—C12—H12	120.5
C3—C4—H4	120.1	C11—C12—H12	120.5
C6—C5—C4	120.3 (2)	C13—C14—C9	119.98 (15)
C6—C5—H5	119.8	C13—C14—H14	120.0
C4—C5—H5	119.8	C9—C14—H14	120.0
C5—C6—C7	120.2 (2)	C14—C13—C12	121.07 (16)
C5—C6—H6	119.9	C14—C13—Cl2	119.29 (14)
C7—C6—H6	119.9	C12—C13—Cl2	119.64 (13)
C6—C7—C2	120.5 (2)		
			
F1—C1—C2—C7	0.8 (3)	C3—N1—C8—C9	179.68 (14)
F3—C1—C2—C7	121.5 (2)	N1—C8—C9—C14	178.07 (15)
F2—C1—C2—C7	−119.7 (2)	N1—C8—C9—C10	−0.4 (2)
F1—C1—C2—C3	−178.7 (2)	C14—C9—C10—O1	−179.21 (14)
F3—C1—C2—C3	−58.0 (3)	C8—C9—C10—O1	−0.7 (2)
F2—C1—C2—C3	60.8 (2)	C14—C9—C10—C11	1.0 (2)
C7—C2—C3—C4	3.8 (3)	C8—C9—C10—C11	179.47 (14)
C1—C2—C3—C4	−176.72 (19)	O1—C10—C11—C12	178.95 (15)
C7—C2—C3—N1	−177.07 (18)	C9—C10—C11—C12	−1.3 (2)
C1—C2—C3—N1	2.5 (3)	O1—C10—C11—Cl1	−1.5 (2)
C8—N1—C3—C4	−44.0 (2)	C9—C10—C11—Cl1	178.29 (12)
C8—N1—C3—C2	136.83 (17)	C10—C11—C12—C13	0.6 (2)
C2—C3—C4—C5	−2.1 (3)	Cl1—C11—C12—C13	−178.95 (13)
N1—C3—C4—C5	178.77 (18)	C10—C9—C14—C13	−0.1 (2)
C3—C4—C5—C6	−0.6 (4)	C8—C9—C14—C13	−178.60 (15)
C4—C5—C6—C7	1.6 (4)	C9—C14—C13—C12	−0.6 (3)
C5—C6—C7—C2	0.1 (4)	C9—C14—C13—Cl2	179.61 (12)
C3—C2—C7—C6	−2.8 (3)	C11—C12—C13—C14	0.4 (3)
C1—C2—C7—C6	177.7 (2)	C11—C12—C13—Cl2	−179.85 (13)

### 2,4-Dichloro-6-{N-[2-(trifluoromethyl)phenyl]carboximidoyl}phenol Hydrogen-bond geometry (Å, º)

**Table:**

*D*—H···*A*	*D*—H	H···*A*	*D*···*A*	*D*—H···*A*
O1—H1···N1	0.82 (2)	1.86 (2)	2.6111 (18)	152 (2)
C14—H14···O1^i^	0.93	2.67	3.515 (2)	151

Symmetry code: (i) *x*+1/2, −*y*+3/2, −*z*+1.
